# Identification of amino acids essential for angulin‐1/3 binding of the tricellular tight junction binder, angubindin‐1

**DOI:** 10.1002/2211-5463.70113

**Published:** 2025-09-10

**Authors:** Taiki Kuzu, Yumi Iwashita, Keisuke Tachibana, Itsuki Nishino, Yuki Niwa, Atsuko Uyeda, Kazuki Matsuo, Masahiro Nagahama, Masuo Kondoh

**Affiliations:** ^1^ Graduate School of Pharmaceutical Sciences The University of Osaka Suita Japan; ^2^ Faculty of Pharmaceutical Sciences Tokushima Bunri University Japan; ^3^ Center for Infectious Disease Education and Research (CiDER) The University of Osaka Japan

**Keywords:** angubindin‐1, angulin‐1/LSR, angulin‐3/ILDR2, tight junction

## Abstract

Tight junctions (TJs) are formed where two or three cells meet and are therefore categorized, respectively, into bicellular TJs (bTJs) and tricellular TJs (tTJs). Angubindin‐1 is the first tTJ modulator enhancing intestinal macromolecule permeation via binding to the key tTJ proteins, angulin‐1 and angulin‐3. It is a fragment (amino acids 421–664) derived from domain IV of *Clostridium perfringens* iota toxin. Here, we identified critical residues (L562, L598, E638, V640, Y643, K644) of angubindin‐1 to be essential for binding to angulins by alanine scanning. Mutants substituting these amino acids with alanine exhibited reduced binding to angulin‐expressing cells. Simultaneous substitution of all these amino acids lost binding to angulins and resulted in the loss of tTJ‐modulating functions of angubindin‐1. These insights highlight crucial residues for the tTJ‐modulating activity of angubindin‐1, which may hold promise in the design of noninvasive, targeted therapeutics using angubindin‐1 as a prototype tTJ modulator to enhance the permeation of drugs.

## Graphical abstract

Angubindin‐1 binds angulin‐1/‐3 at tricellular tight junctions, enhancing intestinal macromolecule permeation. Alanine scanning identified six essential residues (L562, L598, E638, V640, Y643, and K644) of angubindin‐1 critical for binding to angulin‐1/‐3 and permeation‐enhancing activity, providing insights for the development of targeted noninvasive drug delivery strategies.

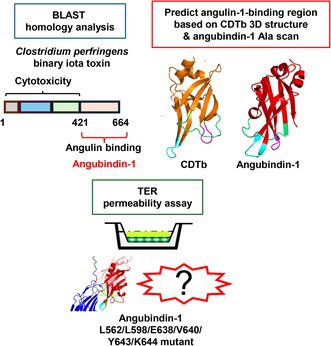

**Abbreviations**
bTJ: bicellular tight junction
CDTb: the binding component of *Clostridium difficile* transferase
FCM: flow cytometry
FD‐4: fluorescein isothiocyanate‐dextran (4 kDa)
GST: glutathione *S*‐transferase
ILDR: immunoglobulin‐like domain‐containing receptor
LSR: lipolysis‐stimulated lipoprotein receptor
TER: transepithelial electric resistance
TJ: tight junction
tTJ: tricellular tight junction

The epithelium functions as a barrier to prevent free movement of a solute from the apical lumen into the body [1]. The intercellular spaces between adjacent epithelial cells are sealed by a meshwork of complex biochemical structures called tight junctions (TJs) [2]. TJs prevent the paracellular free movement of solutes, including drugs, across the epithelium, so modulation of TJ‐based seals in the epithelium is a promising strategy for enhancing the permeation of noninvasive drugs administered to patients [3]. TJs are formed where two or three cells meet and are therefore categorized, respectively, into bicellular TJs (bTJs) and tricellular TJs (tTJs).

The transmembrane proteins that act as key structural and functional components of bTJs and tTJs have been identified: junctional adhesion molecules, occludin, and claudin in bTJs and tricellulin and angulin in tTJs [4]. Claudin comprises a family of 27 members in mammals [5]. Interestingly, the expression profiles and TJ‐sealing functions differ among the 27 claudin members. Thus, claudins have been investigated as targets for enhancing the paracellular permeation of drugs in bTJs [3, 6], and claudin‐1 and claudin‐4 are potential targets for epidermal and mucosal absorption of macromolecules, respectively [7, 8]. A claudin‐4 binder was developed from a toxin fragment to enhance nasal, pulmonary, and intestinal absorption of a biological drug [9], and changes in the claudin specificity of the toxin fragment were used to regulate its mucosal absorption‐enhancing activity [10, 11]. Chemical claudin modulators have enhanced the transdermal granular and intestinal permeation of macromolecules [12, 13].

In comparison to bTJ modulators, the development of tTJ modulators has been slow. Because angulin determines the localization of tricellulin at the tTJ, angulin has more potential as a tTJ modulator than tricellulin [14]. The family of angulins, a type I transmembrane protein, comprises angulin‐1 (also known as lipolysis‐stimulated lipoprotein receptor), angulin‐2 (also known as immunoglobulin‐like domain‐containing receptor 1), and angulin‐3 (also known as immunoglobulin‐like domain‐containing receptor 2) [14, 15, 16]. Although angulin‐1 and angulin‐2 are redundantly expressed in small intestine, colon, bladder, and lung, the expression of angulins is mostly complementary [15, 16]. Our recent analysis of the heterogeneous gene expression of tTJ components in the human intestinal tract revealed that angulin‐3 was very slightly expressed along the intestinal tract, and that expression levels of angulin‐1 and angulin‐2 were higher in the lower tract than in the upper tract [17].

To our best knowledge, angubindin‐1, which binds to angulin‐1 and angulin‐3, is the only tTJ component‐specific modulator [18]. Angubindin‐1 was identified from *Clostridium perfringens* iota toxin, which is a binary toxin consisting of an enzymatic component (Ia) and a receptor‐binding component (Ib) [19]. Knowing that angulin‐1 is a receptor for iota toxin [14, 20], we developed angubindin‐1 as an angulin binder using a fragment of the iota toxin Ib component corresponding to 421–664 amino acids of the C‐terminal domain IV [19]. Angubindin‐1 binds to angulin‐1 and angulin‐3 and thus decreases TJ integrity and enhances jejunal absorption of macromolecules, notably without apparent adverse effects [18]. These findings indicate that an angulin binder is a promising way to enhance the intestinal permeation of drugs, and that modulating the angulin specificity to angubindin‐1 could be a potential strategy for developing tTJ modulators.

Currently, angubindin‐1 is the modulator of angulins, which gives it potential for use as a prototype in the development of an angulin binder that can be used to enhance the permeation of drugs. In this study, we performed functional domain mapping of angubindin‐1 by site‐directed mutagenesis based on alignment analysis of *Clostridium perfringens* iota toxin and *Clostridium difficile* transferase (CDTb), to which angulin‐1 also binds as a receptor and which has a receptor‐binding domain with high similarity to that of iota toxin [20] (Fig. 1). Based on the information acquired in the analysis, we investigated the activity of two mutants to assess the functionality of the putative key amino acids.

**Fig. 1:**
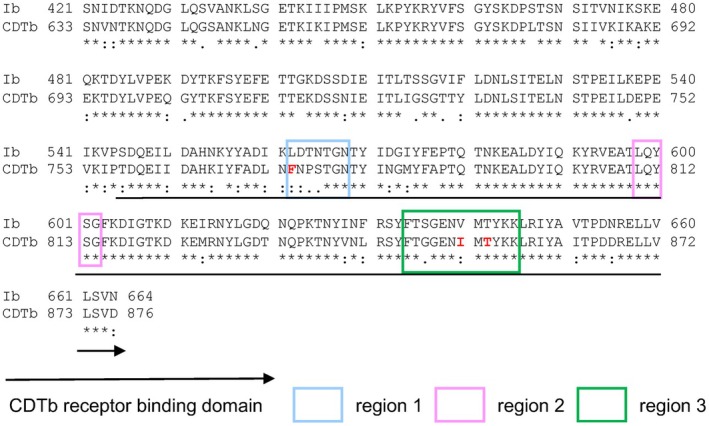
Alignment of amino acid sequences in angubindin‐1 and in the binding component of *Clostridium difficile* transferase (CDTb). The amino acid sequence of the receptor binding component (Ib) of angubindin‐1 (421–664) is shown in the upper row and that of CDTb in the lower row. In this figure, an asterisk (*) indicates positions with a single, fully conserved amino acid residue. A colon (:) denotes conservation among amino acids with strongly similar properties, while a period (.) denotes conservation among amino acids with weakly similar properties. Important regions for receptor binding are framed with squares. The amino acids highlighted in red are considered critical for binding to angulin‐1, as evidenced by the significant reduction in binding affinity observed with D and E substitutions in CDTb. GenBank accession numbers: Ib, WP_003463384; CDTb, WP_102822076. Multiple sequence alignment was performed using CLUSTAL W (https://www.genome.jp/tools‐bin/clustalw) with default parameters.

## Materials and methods

### Reagents

Biotin‐labeled glutathione *S*‐transferase (GST) tag monoclonal antibody (Thermo Fisher Scientific, Waltham, MA, USA; Cat. No. MA4‐004) and Alexa Fluor 647–labeled streptavidin (BioLegend, San Diego, CA, USA; Cat. No. 405237) were purchased, both of research grade.

### Cells

Mouse mammary epithelial EpH4 cells (designated as angulin‐1‐expressing EpH4) and angulin‐1‐knockdown EpH4 cells with overexpression of FLAG‐tagged angulin‐3 were kindly provided by Dr. M. Furuse (Kobe University, Japan) [15]. All cells were maintained in Dulbecco's modified Eagle's medium (DMEM) supplemented with 10% fetal bovine serum in a 5% CO_2_ atmosphere at 37 °C. Caco‐2 cell lines (RCB0988, RRID: CVCL_0025) used in this study were obtained from RIKEN BRC Cell Bank (Tsukuba, Japan). Caco‐2 cells were maintained in modified Eagle's medium (MEM) supplemented with 10% fetal bovine serum and 1% MEM Non‐Essential Amino Acids Solution (Nacalai Tesque, Kyoto, Japan) in a 5% CO_2_ atmosphere at 37 °C. Caco‐2 cells were authenticated by short tandem repeat profiling by the provider (RIKEN BRC Cell Bank) and confirmed to match the official reference profiles. We used the cells for experiments within 2 months after thawing. The absence of mycoplasma contamination was verified by MycoAlert Mycoplasma Detection Kit (Lonza, Basel, Switzerland).

### BLAST search

The protein blast program available from NCBI (http://www.ncbi.nlm.nih.gov/BLAST/) was used to search for amino acid sequences similar to the query sequence, aiming to find good alignments between the query sequence and database sequences [21]. Multiple sequence alignment and amino acid similarity were elucidated by running clustal w [22].

### Preparation of angubindin‐1 and angubindin‐1 mutants

GST‐tagged angubindin‐1 was purified according to a previous report with slight modification [23]. The expression plasmids in which angubindin‐1 had substitutions to alanine, aspartic acid, or glutamic acid were prepared by mutagenesis using KOD Plus (Toyobo, Osaka, Japan) with forward and reverse primers (Table S1). In the generated mutants of angubindin‐1, the following residues were substituted to alanine: leucine at positions 562 (L562A) and 598 (L598A), glutamic acid at position 638 (E638A), valine at position 640 (V640A), tyrosine at position 643 (Y643A), and lysine at position 644 (K644A).

For alanine scanning experiments, angubindin‐1 and various angubindin‐1 mutants were expressed and purified. Briefly, *E. coli* strain BL21(DE3) (Sigma‐Aldrich, St. Louis, MO, USA) was transformed with expression plasmids, and recombinant proteins were induced by treatment with isopropyl β‐d‐thiogalactopyranoside (Nacalai Tesque). The protein was purified by using GST SpinTrap (Cytiva, Marlborough, MA, USA). GST‐tagged angubindin‐1 and angubindin‐1 mutants were eluted using an elution buffer [50 mm Tris/HCl (pH 9.0), 10 mm reduced glutathione]. Eluted protein was concentrated by using an Amicon Ultra‐4 10 K filter (Merck Millipore, Burlington, MA, USA), and then, the buffer was exchanged to PBS using a PD‐10 column (Cytiva). The protein concentration was determined by using Protein Assay BCA Kit (Nacalai Tesque).

For transepithelial electrical resistance (TER) measurement and the permeability assay, an angubindin‐1 mutant with all six alanine substitutions (6A mutant) and an angubindin‐1 mutant with substitutions of leucine to aspartic acid at position 562 (L562D), L598A, E638A, valine to glutamic acid at position 640 (V640E), Y643A, and K644A (4ADE mutant) were purified as described previously with some modification [23]. Briefly, *E. coli* strain BL21(DE3) was transformed with expression plasmids for angubindin‐1, angubindin‐1 6A mutant, or angubindin‐1 4ADE mutant, and the protein expression was induced by treatment with isopropyl β‐d‐thiogalactopyranoside. GST‐tagged angubindin‐1 was purified using glutathione sepharose resin (Cytiva). Thrombin (Nacalai Tesque) was treated to cleave the GST‐tagged proteins bound to the glutathione sepharose resin to obtain untagged angubindin‐1, and the elute was collected. Thrombin was removed by treatment of the elute with Benzamidine Sepharose 4 Fast Flow resin (Cytiva). The eluted protein then underwent the same steps as those for the individual mutants.

### Flow cytometry analysis

For flow cytometry analysis, cells were collected and treated with 1% BSA‐PBS for 30 min on ice. GST‐angubindin‐1 (final 10 μg·mL^−1^) was added and incubated on ice for 1 h. The cells were washed with 1% BSA‐PBS and subsequently treated with biotinylated GST‐tag monoclonal antibody on ice for 30 min. After washing with 1% BSA‐PBS, Alexa Fluor 647‐conjugated streptavidin was added, and the cells were incubated on ice for 20 min under shading. After washing with 0.2% BSA‐PBS, the cells were treated with propidium iodide (Miltenyi Biotec, Bergisch Gladbach, Germany). The antibody‐bound cells were detected with MACSQuant X (Miltenyi Biotec) using flowjo v10.8.1 (BD Biosciences, Franklin Lakes, NJ, USA).

### Measurement of TER and permeability assay

Cell culture inserts were placed over 24‐well plates (Corning, NY, USA, Transparent PET Membrane, 0.4 μm, 24‐well); then, cells were seeded in the top wells at a density of 3 × 10^4^ cells/300 μL, and 700 μL of DMEM was added to the bottom wells. The cells were cultured at 37 °C in the presence of 5% CO_2_, and TER was monitored every 2–3 days using a voltohmmeter (Millicell‐ERS‐2, Merck Millipore). When the TER reached a plateau, tTJ modulators were added to the bottom wells at final concentrations of 30, 100, and 300 μg·mL^−1^ (10% v/v) and, as a blank and negative control, 700 μL (10% v/v) PBS‐containing DMEM was added to a separate bottom well. Next, 300 μL of DMEM was added to the top wells. The TER value was measured immediately before adding the reagent, and this was taken as the value at 0 h. The TER value was measured every hour after adding tTJ modulators. After 24‐h treatment, 200 μL of 0.1 mg·mL^−1^ fluorescein (Sigma‐Aldrich) or 10 mg·mL^−1^ fluorescein isothiocyanate‐dextran (4 kDa) (FD‐4) (Sigma‐Aldrich) dissolved in DMEM without phenol red was added to the top well for tracer flux measurement. After 2 h, an aliquot of the medium in the bottom well was collected and measured using a fluorescence spectrophotometer (TriStar LB941, Berthold Technologies, Bad Wildbad, Germany) at an excitation/emission wavelength of 485/535 nm. The content of fluorescein or FD‐4 was determined by extrapolation from the standard curve of fluorescein or FD‐4 concentration using linear regression.

### Statistical analysis

Statistical analysis of the data was carried out using graphpad prism 9.4.1 software (GraphPad Software, Boston, MA, USA). Data are expressed as the mean ± standard error of the mean (SEM) from 4 to 8 replicates, and Student's *t*‐test or one‐way ANOVA with Dunnett's multiple comparison test was used to analyze statistical significance.

## Results

### Estimation of the angulin‐1 binding site of angubindin‐1

Because the structure of angubindin‐1 remains undetermined, we sought to infer its angulin‐binding site by examining structural similarities with related proteins. Therefore, we performed a BLAST search based on the amino acid sequence of angubindin‐1, namely binary iota toxin binding subunit Ib 421–664 (GenBank protein accession: WP_003463384), and found that the amino acid sequence of angubindin‐1 was most similar to that of the binding component of CDTb (GenBank protein accession: WP_102822076), whose crystal structure has been solved (PDBID: 6UWO) [24]. In a total of 244 amino acids, 197 amino acids (80.7%) were identical to CDTb (Fig. 1). CDTb binds to angulin‐1, and its binding domain is in the region of amino acids 757–876 (Fig. 1) [24, 25]. Furthermore, the CDTb conformation has also been determined as having three loop regions that interact with angulin‐1 [20, 24]. A detailed analysis of the sequence of angubindin‐1 that corresponds to the three loop regions of CDTb revealed that 3 of the 7 amino acids (42.8%) in region 1, all 5 amino acids (100%) in region 2, and 10 of 12 amino acids (83.3%) in region 3 matched (Fig. 1). Thus, we focused on these loop regions of angubindin‐1 for further investigation.

### Alanine scanning for disruption of angubindin‐1 binding to angulin‐1 and angulin‐3

**Fig. 2:**
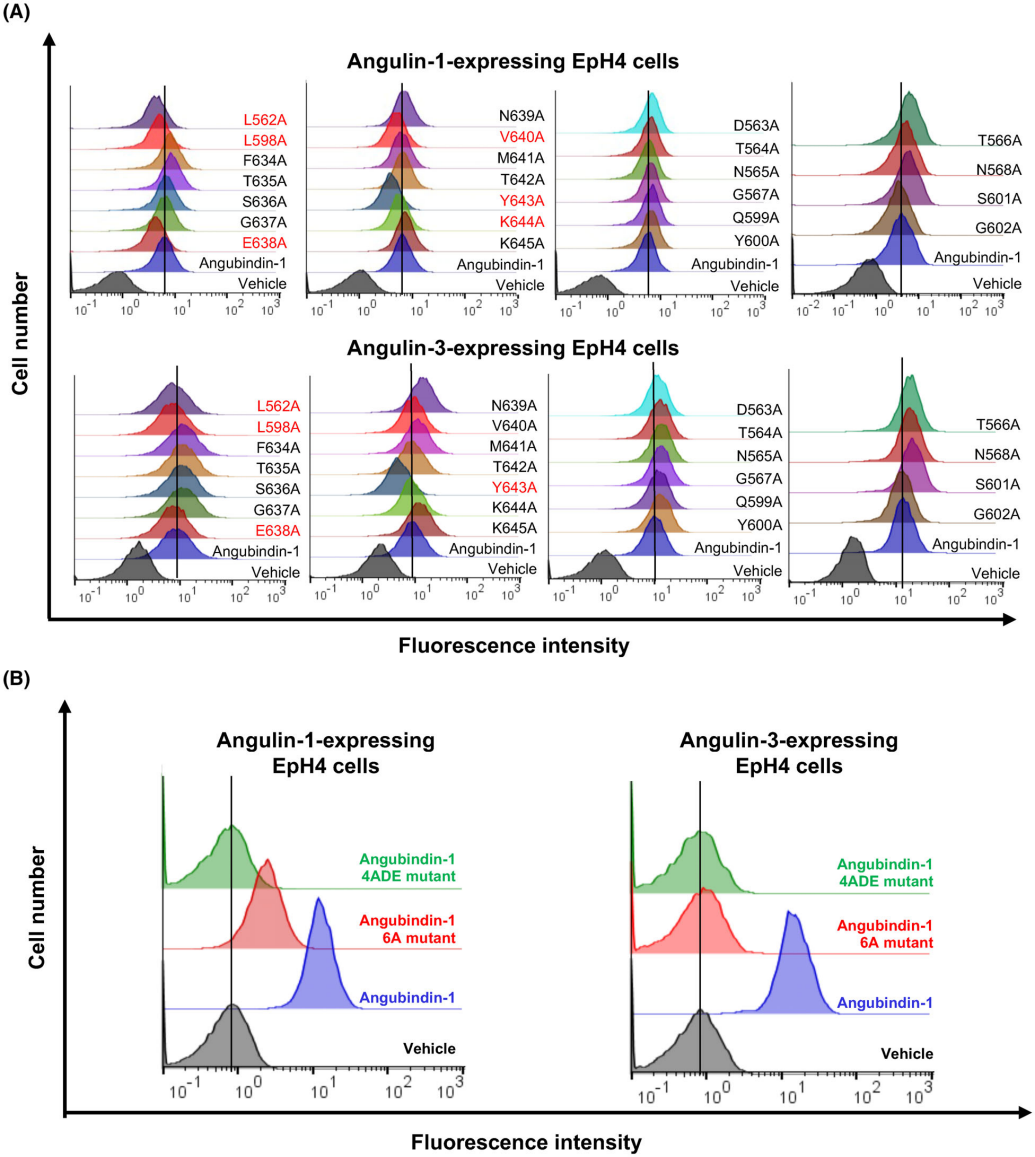
Alanine scan of presumed binding sites in angubindin‐1. (A, B) EpH4 cells expressing angulin‐1 or angulin‐3 were treated with vehicle (PBS), GST‐angubindin‐1, or GST‐angubindin‐1 mutants (10 μg·mL^−1^) for 1 h at 4 °C. Then, GST‐angubindin‐1‐bound cells were detected by biotin‐conjugated anti‐GST‐tagged antibody and Alexa Fluor 647‐conjugated streptavidin, and the labeled cells were detected by flow cytometry analysis. (A) Mutants with alanine substitutions. Angulin‐1‐expressing EpH4 cells demonstrated reduced binding activity in the L562A, L598A, E638A, V640A, Y643A, and K644A mutants, and angulin‐3‐expressing EpH4 cells demonstrated reduced binding activity in the L562A, L598A, E638A, and Y643A mutants. (B) A mutant with all six substitutions to alanine (6A) and the 6A mutant with further substitutions of L562D and V640E (4ADE). The 6A mutant completely lost binding to angulin‐3 but retained weak binding activity to angulin‐1. In contrast, the 4ADE mutant exhibited a complete loss of binding to both angulin‐1 and angulin‐3. The data are representative of biologically independent experiments (*n* = 3).

We utilized our homology analysis with CDTb to design angubindin‐1 mutants by substituting specific amino acids in regions 1–3 with alanine and then used flow cytometry to assess the binding activities of these mutants to EpH4 cells expressing angulin‐1 or angulin‐3. As shown in Fig. 2A, our findings indicated that the binding activity of angubindin‐1 to angulin‐1 cells decreased in the L562A mutant (which had a substitution in region 1), the L598A mutant (which had a substitution in region 2), and the E638A, V640A, Y643A, and K644A mutants (which had substitutions in region 3). In addition, the L562A, L598A, E638A, and Y643A mutants also had decreased binding to angulin‐3. Notably, the Y643A mutation significantly diminished the binding to both angulin‐1 and angulin‐3. Interestingly, the F634A, T635A, T566A, N568A, and S601A mutants improved the binding to angulin‐1, and the F634A, T635A, S636A, N639A, M641A, K645A, T564A, N565A, G567A, Q599A, Y600A, T566A, N568A, and S601A mutants showed higher binding to angulin‐3.

To further understand the critical residues for binding of angubindin‐1 to angulin, we focused on the L562, L598, E638, V640, Y643, and K644 because these mutants attenuated angulin‐1 binding, and angulin‐1 is a potent target for mucosal permeation enhancer [17]. We studied the effect of the 6A mutant, which had all six amino acid substitutions. The 6A mutant weakened angulin‐1 activity and abolished angulin‐3 binding activity, respectively (Fig. 2B).

It has been reported that mutations F774D, I852E, and T854E in CDTb significantly reduce binding activity to angulin‐1 [24]. Since the 6A mutant still had weak binding activity to angulin‐1 (Fig. 2B), we tried to mutate the 6A mutant with reference to the above finding in CDTb. By sequence alignment analysis, F774, I852, and T854 of CDTb correspond to L562, V640, and T642 of angubindin‐1, respectively (Fig. 1). Notably, two of these three amino acids (L562 and V640) were experimentally demonstrated to be important amino acids for binding to angulins (Fig. 2A). Therefore, the 6A mutant was additionally mutated with L562D and V640E to generate the 4ADE mutant. Introduction of L562D and V640E mutations in the 6A mutant resulted in the loss of binding to both angulin‐1 and angulin‐3 (Fig. 2B).

### Effects of 6A and 4ADE mutants on the barrier function of angulin

**Fig. 3:**
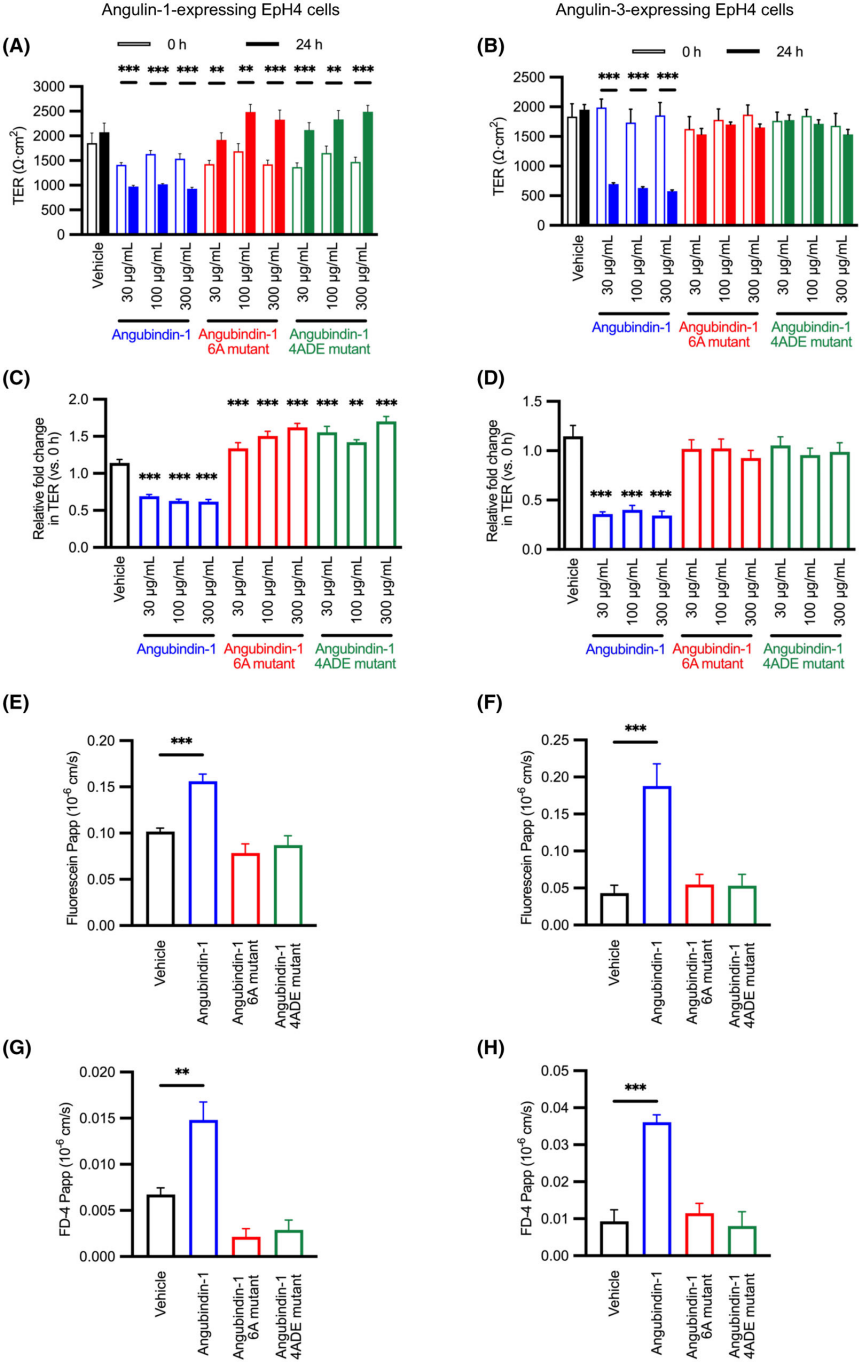
Effect of angubindin‐1 and angubindin‐1 mutants on the epithelial barrier. (A–D) Angulin‐1‐expressing EpH4 cells or angulin‐3‐expressing EpH4 cells were seeded on transwell cell culture inserts. After the electrical resistance values had stabilized, angubindin‐1 or a mutant of angubindin‐1 with six alanine substitutions (6A) or a mutant with further substitutions in 6A of L562D and V640E (4ADE) was added to the bottom side, and cells were treated for 24 h. The change in transepithelial electric resistance (TER) after 24‐h treatment was monitored (A, B) (*n* = 8). Relative TER to the values at 0 h was calculated (C, D). A reduction in TER was observed in both angulin‐1 and angulin‐3‐expressing cells following treatment with angubindin‐1. In contrast, treatment with the 6A or 4ADE mutants did not result in a reduction of TER. (E‐H) The permeability to fluorescein (332 Da) (E, F) (*n* = 6) and to fluorescein isothiocyanate‐dextran (4 kDa) (FD‐4) (G, H) (*n* = 4) was assessed after 24 h treatment with angubindin‐1 and its mutants (100 μg·mL^−1^). The permeability to fluorescein and FD‐4 was significantly increased after 24‐h treatment with angubindin‐1. Conversely, neither the 6A nor the 4ADE mutants affected the barrier function. **P < 0.01 and ***P < 0.001 by one‐way ANOVA with Dunnett's multiple comparison test vs. vehicle. All values are shown as mean ± SEM.

Next, we evaluated the effects of 6A and 4ADE mutants on barrier function by measuring TER and permeability in cells expressing angulin‐1 or angulin‐3. In angulin‐1‐expressing cells, 24‐h treatment with angubindin‐1 at 30 and 100 μg·mL^−1^ reduced the TER to 69.2% and 61.9% of that of the cells treated with vehicle, respectively, but there was no decrease in TER in the cells treated with 6A mutant or 4ADE mutant at 300 μg·mL^−1^ (Fig. 3A,C). Similarly, in the angulin‐3‐expressing cells, TER decreased after 24‐h treatment with angubindin‐1 at 30 and 100 μg·mL^−1^ to 36.0% and 40.2% of that of the vehicle group, respectively, but there was no decrease in TER in the cells treated with 6A mutant or 4ADE mutant at 300 μg·mL^−1^ (Fig. 3B,D).

FD‐4 is a paracellular flux marker [8]. Strokes radius of FD‐4 is calculated to be 1.4 nm. The cavity of TJ is estimated to be 0.5 nm in physiological condition, and a paracellular permeation enhancer opened TJ up to 1.5 nm [8, 26, 27]. Angubindin‐1 treatment increased paracellular permeability for FD‐4 [18]. Therefore, we investigated the effects of the 6A mutant and 4ADE mutant on paracellular permeability using FD‐4 (4 kDa) and fluorescein (332 Da). Treatment with angubindin‐1 of cells expressing angulin‐1 or angulin‐3 increased the permeability to fluorescein and FD‐4 compared with the vehicle group; however, there was no increase in the permeability for fluorescein and FD‐4 by treatment with the 6A mutant and 4ADE mutant (Fig. 3E–H).

### Effects of 6A and 4ADE mutants on the barrier function in human intestinal cells

**Fig. 4:**
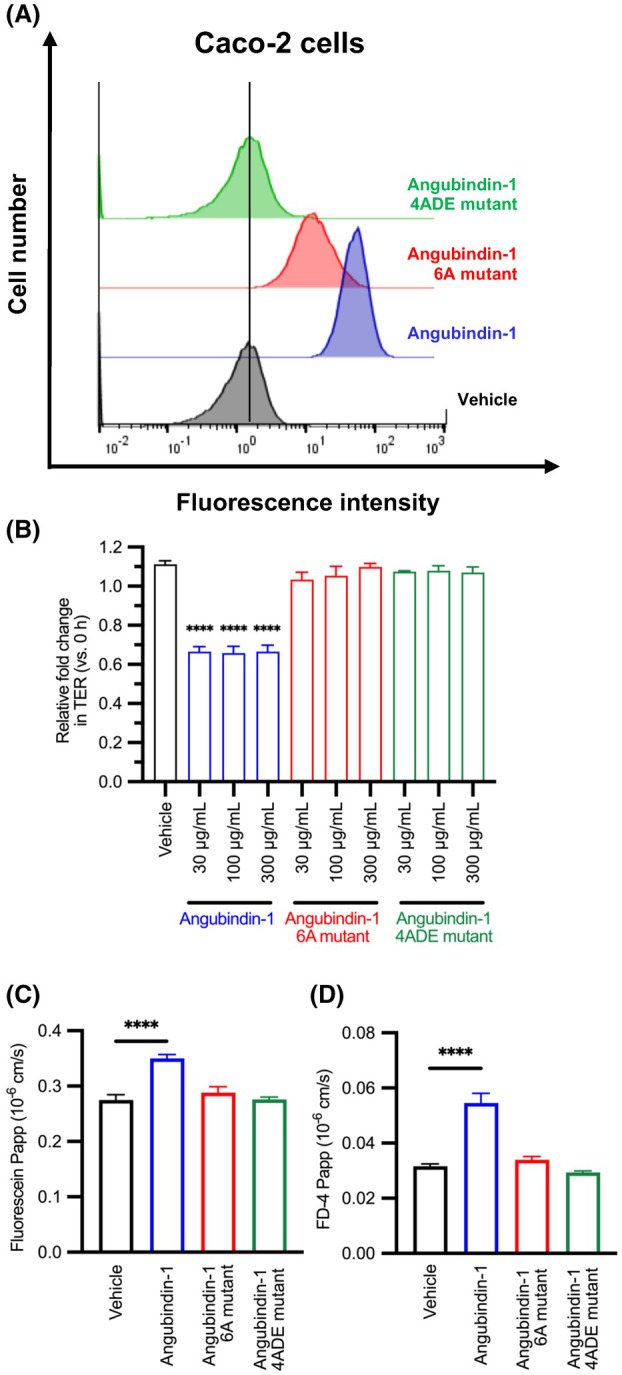
Effect of angubindin‐1 and angubindin‐1 mutants in Caco‐2 cells. (A) Flow cytometry analysis of cells treated with angubindin‐1 or with a mutant of angubindin‐1 with six alanine substitutions (6A) or a mutant with further substitutions in 6A of L562D and V640E (4ADE). Caco‐2 cells were treated with vehicle (PBS), GST‐angubindin‐1, or GST‐angubindin‐1 6A or 4ADE mutants (10 μg·mL^−1^) for 1 h at 4 °C. Then, bound cells were detected by biotin‐conjugated anti‐GST‐tagged antibody and Alexa Fluor 647‐conjugated streptavidin, and labeled cells were analyzed. The 6A mutant retained weak binding activity in Caco‐2 cells. In contrast, the 4ADE mutant exhibited a complete loss of binding in Caco‐2 cells. The data are representative of biologically independent experiments (*n* = 3). (B) Caco‐2 cells were treated for 24 h with angubindin‐1, angubindin‐1 6A mutant, or angubindin‐1 4ADE mutant (30–300 μg·mL^−1^). The change in transepithelial electric resistance (TER) after 24‐h treatment relative to the values at 0 h was calculated (*n* = 3). A reduction in TER was observed in Caco‐2 cells following treatment with angubindin‐1. In contrast, no changes in TER were observed with the 6A or 4ADE mutants. (C, D) The permeability to fluorescein (332 Da) (C) and fluorescein isothiocyanate‐dextran (4 kDa) (FD‐4) (D) was assessed after 24‐h treatment of angubindin‐1 and mutants (100 μg·mL^−1^) (*n* = 5). The permeability to fluorescein and FD‐4 was significantly increased after 24‐h treatment of angubindin‐1. Conversely, none of the 6A or 4ADE mutants affected the barrier function. ****P < 0.0001 by one‐way ANOVA with Dunnett's multiple comparison test vs. vehicle. All values are shown as mean ± SEM.

Finally, we investigated the effects of our angubindin‐1 mutants in an *in vitro* human intestinal permeation model, Caco‐2 monolayer cell sheet [28, 29]. Angubindin‐1 bound to Caco‐2 cells, but 6A mutant binding decreased and 4ADE mutant binding was lost (Fig. 4A). Similar to the results found in EpH4 angulin‐expressing cells, treatment of Caco‐2 cells with angubindin‐1 decreased TER (Fig. 4B) and increased permeation of fluorescein and FD‐4 compared to those of vehicle treatment (Fig. 4C,D). However, 6A mutant and 4ADE mutant attenuated the permeation‐enhancing activity (Fig. 4C,D). Thus, L562, L598, E638, V640, Y643, and K644 might be key amino acid residues for the tTJ‐modulating activity of angubindin‐1 in a human intestine.

## Discussion

*Clostridium perfringens* iota toxin, *C. difficile* toxin, and *C. spiroforme* toxin are binary toxins that each consist of a cytotoxic Ia domain and a receptor‐binding Ib domain [30]. A receptor for these toxins is angulin‐1 [31]. Previous structural analysis of the Ib domain of *Clostridium difficile* toxin revealed that the bottom face of domain VI of the Ib domain may be involved in its binding to angulin‐1 and that the residues Phe774, Ile852, and Thr854 located in the bottom face are key amino acids for angulin‐1 binding [24]. These positions in CDTb respectively correspond to Leu562, Val640, and Thr642 in angubindin‐1. Our substitution of Leu562 or Val640 with alanine reduced the binding of angubindin‐1 to angulin‐1. Thus, Leu562 and Val640 are key residues for the binding of angubindin‐1 to angulin‐1.

Monomeric Ib domain of iota toxin binds to angulin‐1, and then the resulting complex is oligomerized to produce the oligomeric prepore state [32]. Treatment of cells with angubindin‐1 revealed that angulin‐bound angubindin‐1 moves to lipid rafts and undergoes oligomerization in the cell membrane [33]. Phe774 of CDTb is involved in its binding to angulin‐1 and oligomerization of CDTb to a heptamer [24]. In the same study, substitution of Phe with Asp at position 774 decreased the binding of CDTb to angulin‐1, indicating that hydrophobic interaction with angulin‐1 via Phe774 may be involved in the interaction of angulin‐1 with CDTb. In our study, replacement of Leu by Asp at position 562 similarly decreased the binding of angubindin‐1 to angulin‐1. Thus, hydrophobic interaction at Leu562 may be associated with the binding of angubindin‐1 to angulin‐1 and its subsequent oligomerization. In turn, this interaction and subsequent oligomerization may prevent further recruitment of monomeric angulin‐1 to the tricellular contact and subsequent formation of tTJ seals.

Some single alanine‐substituted mutants in our study appeared to increase interaction with angulin‐1 or angulin‐3 (Fig. 2A). The binding domain of angulin‐1 to Ib is 15 amino acids in its N‐terminal [34]. In angulin‐1, ‐2, and ‐3, these 15 amino acids share only one amino acid identity (MAPAA SACAG APGSH in angulin‐1, Protein accession number: AK136284; MGCGL LAAGL LLFTW in angulin‐2, AK136284; MDRVV LGWTA VFWLT in angulin‐3, FJ024498) [15]. Four out of five of the mutants with increased interaction with angulin‐1 replace hydrophilic amino acids with hydrophobic alanine. Ten of the 14 alanine mutants with enhanced binding to angulin‐3 have hydrophilic amino acids substituted with alanine. As the sequences given for the N‐terminals of angulins above show, most of the 15 N‐terminal amino acids are hydrophobic in the putative binding domain of angulin‐1 to CDTb [34]. The three loop regions, in which amino acids were replaced by alanine, are in the putative angulin‐1 binding domain [24]. Thus, a possible explanation for enhanced angulin binding in some single alanine‐substituted mutants may be hydrophobic interaction based on the remaining interactions with angubindin‐1 via the three loop regions in angulin.

Although the 6A and 4ADE mutants attenuated and lost binding to angulin‐1 and angulin‐3, respectively, treatment with the 6A mutant or the 4ADE mutant increased the TER values in angulin‐1 expressing cells, but not in angulin‐3 expressing cells. This discrepancy may be due to the difference between flow cytometry analysis and TER analysis. A cell suspension was used for flow cytometry analysis, and therefore, the interaction of the mutants with the extracellular loop domain on the cell was evaluated. In the monolayer cell sheet used for TER analysis, the extracellular loop region may not have been exposed; therefore, the mutants may have interacted with angulin‐1 contained in the central tube of the tTJ at sites other than the extracellular loop region. The interaction of the mutants with a different region of angulin‐1 may be a hydrophobic interaction.

In summary, our analysis of a series of alanine substitutions revealed that 6A mutants attenuated and lost binding to angulin‐1 and angulin‐3, respectively. This suggests that the interaction of angubindin‐1 to angulin‐1 and its resulting prevention of the tTJ‐barrier function may be different from that to angulin‐3. 6A mutant and 4ADE mutant treatment increased the TER value in angulin‐1‐expressing cells but not angulin‐3‐expressing cells. Although whole proteins of angulin‐1 and angulin‐3 share 33% amino acid identity [15], angulin‐3 was originally identified as a protein expressed in lymphoma cells [35], and the expression of angulin‐3, but not angulin‐1, was observed in the brain, eye, heart, and skeletal muscle in mice [15]. Angulin‐1 expression was higher than angulin‐3 expressed in gastrointestinal tract in mice and humans [15, 17]. A reduction of affinity to angulin‐3 without a decrease in angulin‐1 binding would be a method to generate safe and efficient intestinal permeation enhancers that can modulate tTJ‐seals. Angulin‐2 was expressed in the colon and rectum in the human gastrointestinal tract [17]. Angulin‐2 modulator might be a permeation enhancer for suppository. Angubindin‐1 can serve as a prototype tTJ modulator. We will make a phage display library of angubindin‐1 mutating functional amino acids involved in its angulin‐binding. We will also investigate angulin‐binding activity and tight junction‐modulating activity of novel angulin binders, screened among the angubindin‐1 mutated library. In this study, we provided functional domain mapping data on the binding of angubindin‐1 to angulin and investigated its modulation of angulin‐based TJ integrity. These findings will be useful in the development of a tTJ‐targeted intestinal permeation enhancer by using angubindin‐1 as a lead tTJ modulator for noninvasive pharmaceutical therapies.

## Conflict of interest

The authors declare no conflict of interest.

## Author contributions

TK contributed to the investigation, data curation, formal analysis, methodology, validation, visualization, writing—original draft. YI contributed to the conceptualization, investigation, methodology, validation, writing—original draft, review, and editing. KT contributed to the supervision, conceptualization, data curation, methodology, supervision, writing—review and editing. IN contributed to the investigation, validation, writing—review and editing. YN contributed to the data curation, methodology, writing—review and editing. AU contributed to the investigation, validation, writing—review and editing. KM contributed to the investigation, writing—review and editing. MN contributed to the resources, writing—review and editing. MK contributed to the conceptualization, data curation, funding acquisition, project administration, supervision, writing—review and editing. All authors read and approved the manuscript.

## Supporting information


**Table S1.** List of primers used for angubindin‐1 mutagenesis.

## Data Availability

The data that support the findings of this study are available from the corresponding author [masuo@phs.osaka-u.ac.jp] upon reasonable request.
