# Supplementary material for: Identification of amino acids essential for angulin‐1/3 binding of the tricellular tight junction binder, angubindin‐1
Source: FEBS Open Bio. 2025 Sep 10;16(1):116–26. doi: 10.1002/2211-5463.70113 (PMC12767772; doi:10.1002/2211-5463.70113)
Supplement: Supplementary file 1 — Table S1. List of primers used for angubindin‐1 mutagenesis. [file FEB4-16-116-s001.docx]

Supplementary Table 1. Angubindin-1 mutagenesis primer list

| Mutant | Primer name | Sequence (5' to 3') | Template |
| --- | --- | --- | --- |
| L562A | L562A-fw | CAAATATTATGCAGATATAAAGGCTGACACAAATACAGGAAA | pGEX-Ib421-664    pGEX-Ib421-664 |
|  | L562A-rv | TTTCCTGTATTTGTGTCAGCCTTTATATCTGCATAATATTTG |  |
| D563A | D563A-fw | TGCAGATATAAAGCTTGCCACAAATACAGGAAACAC |  |
|  | D563A-rv | GTGTTTCCTGTATTTGTGGCAAGCTTTATATCTGCA |  |
| T564A | T564A-fw | GCAGATATAAAGCTTGACGCAAATACAGGAAACACTTA |  |
|  | T564A-rv | TAAGTGTTTCCTGTATTTGCGTCAAGCTTTATATCTGC |  |
| N565A | N565A-fw | GATATAAAGCTTGACACAGCTACAGGAAACACTTATATAG |  |
|  | N565A-rv | CTATATAAGTGTTTCCTGTAGCTGTGTCAAGCTTTATATC |  |
| T566A | T556A-fw | AAGCTTGACACAAATGCAGGAAACACTTATATAG |  |
|  | T566A-rv | CTATATAAGTGTTTCCTGCATTTGTGTCAAGCTT |  |
| G567A | G567A-fw | CTTGACACAAATACAGCGAACACTTATATAGATGG |  |
|  | G567A-rv | CCATCTATATAAGTGTTCGCTGTATTTGTGTCAAG |  |
| N568A | N568A-fw | TGACACAAATACAGGAGCCACTTATATAGATGGTATATATT |  |
|  | N568A-rv | AATATATACCATCTATATAAGTGGCTCCTGTATTTGTGTCA |  |
| L598A | L598A-fw | ATAGAGTTGAAGCAACTGCGCAATATTCAGGATTTA |  |
|  | L598A-fw | TAAATCCTGAATATTGCGCAGTTGCTTCAACTCTAT |  |
| Q599A | Q599A-fw | GAGTTGAAGCAACTTTGGCATATTCAGGATTTAAAGATAT |  |
|  | Q599A-rv | ATATCTTTAAATCCTGAATATGCCAAAGTTGCTTCAACTC |  |
| Y600A | Y600A-fw | TGAAGCAACTTTGCAAGCTTCAGGATTTAAAGATATTG |  |
|  | Y600A-rv | CAATATCTTTAAATCCTGAAGCTTGCAAAGTTGCTTCA |  |
| S601A | S601A-fw | GCAACTTTGCAATATGCAGGATTTAAAGATATTG |  |
|  | S601A-rv | CAATATCTTTAAATCCTGCATATTGCAAAGTTGC |  |
| G602A | G602A-fw | CTTTGCAATATTCAGCATTTAAAGATATTGGAAC |  |
|  | G602A-rv | GTTCCAATATCTTTAAATGCTGAATATTGCAAAG |  |
| F634A | F634A-fw | TTATATTAATTTTAGAAGTTATGCTACTAGTGGAGAAAATG |  |
|  | F634A-rv | CATTTTCTCCACTAGTAGCATAACTTCTAAAATTAATATAA |  |
| T635A | T635A-fw | TAATTTTAGAAGTTATTTTGCTAGTGGAGAAAATG |  |
|  | T635A-rv | CATTTTCTCCACTAGCAAAATAACTTCTAAAATTA |  |
| S636A | S636A-fw | TAATTTTAGAAGTTATTTTACTGCTGGAGAAAATGTTATG |  |
|  | S636A-rv | CATAACATTTTCTCCAGCAGTAAAATAACTTCTAAAATTA |  |
| G637A | G637A-fw | GAAGTTATTTTACTAGTGCAGAAAATGTTATGACA |  |
|  | G637A-rv | TGTCATAACATTTTCTGCACTAGTAAAATAACTTC |  |
| E638A | E638A-fw | GTTATTTTACTAGTGGAGCAAATGTTATGACATATAA |  |
|  | E638A-rv | TTATATGTCATAACATTTGCTCCACTAGTAAAATAAC |  |
| N639A | N639A-fw | GTTATTTTACTAGTGGAGAAGCTGTTATGACATATAAAAAAT |  |
|  | N639A-rv | ATTTTTTATATGTCATAACAGCTTCTCCACTAGTAAAATAAC |  |
| V640A | V640A-fw | TACTAGTGGAGAAAATGCTATGACATATAAAAAATTAAG |  |
|  | V640A-rv | CTTAATTTTTTATATGTCATAGCATTTTCTCCACTAGTA |  |
| M641A | M641A-fw | TAGTGGAGAAAATGTTGCGACATATAAAAAATTAAGA |  |
|  | M641A-rv | TCTTAATTTTTTATATGTCGCAACATTTTCTCCACTA |  |
| T642A | T642A-fw | GTGGAGAAAATGTTATGGCATATAAAAAATTAAGAATATATG |  |
|  | T642A-rv | CATATATTCTTAATTTTTTATATGCCATAACATTTTCTCCAC |  |

| Y643A | Y643A-fw | TGGAGAAAATGTTATGACAGCTAAAAAATTAAGAATATATGCAG | pGEX-Ib421-664 |
| --- | --- | --- | --- |
|  | Y643A-rv | CTGCATATATTCTTAATTTTTTAGCTGTCATAACATTTTCTCCA |  |
| K644A | K644A-fw | GAGAAAATGTTATGACATATGCAAAATTAAGAATATATGCAG |  |
|  | K644A-rv | CTGCATATATTCTTAATTTTGCATATGTCATAACATTTTCTC |  |
| K645A | K645A-fw | GAAAATGTTATGACATATAAAGCATTAAGAATATATGCAGTTAC |  |
|  | K645A-rv | GTAACTGCATATATTCTTAATGCTTTATATGTCATAACATTTTC |  |
| Y643A/K644A | K644A_Y643A-fw | GGAGAAAATGTTATGACAGCTGCAAAATTAAGAATATATG | pGEX-Ib421-664_K644A |
|  | K644A_Y643A-rv | CATATATTCTTAATTTTGCAGCTGTCATAACATTTTCTCC |  |
| V640A/Y643A/K644A | Y643A/K644A_V640A-fw | TTACTAGTGGAGAAAATGCTATGACAGCTGCAAA | pGEX-Ib421-664_Y643A/K644A |
|  | Y643A/K644A_V640A-rv | TTTGCAGCTGTCATAGCATTTTCTCCACTAGTAA |  |
| E638A/V640A/Y643A/K644A | V640A/Y643A/K644A_E638A-fw | GAAGTTATTTTACTAGTGGAGCAAATGCTATGACAGCTG | pGEX-Ib421-664_V640A/Y643A/V644A |
|  | V640A/Y643A/K644A_E638A-rv | CAGCTGTCATAGCATTTGCTCCACTAGTAAAATAACTTC |  |
| L598A/E638A/V640A/Y643A/K644A | L598A-fw | ATAGAGTTGAAGCAACTGCGCAATATTCAGGATTTA | pGEX-Ib421-664  _E638A/V640A/Y643A/K644A |
|  | L598A-rv | TAAATCCTGAATATTGCGCAGTTGCTTCAACTCTAT |  |
| L562A/L598A/E638A/V640A/Y643A/K644A | L562A-fw | CAAATATTATGCAGATATAAAGGCTGACACAAATACAGGAAA | pGEX-Ib421-664  _L598A/E638A/V640A/Y643A/K644A |
|  | L562A-rv | TTTCCTGTATTTGTGTCAGCCTTTATATCTGCATAATATTTG |  |
| L562A/L598A/E638A/V640E/Y643A/K644A | V640E_fw | TTACTAGTGGAGCAAATGAGATGACAGCTGCAAAAT | pGEX-Ib421-664  _L562A/L598A/E638A/V640A/Y643A/  K644A |
|  | V640E_rv | ATTTTGCAGCTGTCATCTCATTTGCTCCACTAGTAA |  |
| L562D/L598A/E638A/V640E/Y643A/K644A | L562D_fw | AATATTATGCAGATATAAAGGATGACACAAATACAGGAA | pGEX-Ib421-664_L562A/L598A  /E638A/V640E/Y643A/K644A |
|  | L562D_rv | TTCCTGTATTTGTGTCATCCTTTATATCTGCATAATATT |  |
